# Mental health and life satisfaction among those advised to shield during the COVID-19 pandemic in the UK: a secondary analysis of the Understanding Society longitudinal study

**DOI:** 10.3389/fpubh.2023.1235903

**Published:** 2023-09-27

**Authors:** Simon George Morris, Laura Kudrna, James Martin

**Affiliations:** ^1^Institute of Applied Health Research, University of Birmingham, Birmingham, United Kingdom; ^2^Public Health Specialty Training Programme, NHS England - West Midlands, Birmingham, United Kingdom

**Keywords:** mental health, life satisfaction, wellbeing, shielding, clinically extremely vulnerable, COVID-19 pandemic

## Abstract

**Introduction:**

During the COVID-19 pandemic in the UK, those considered most vulnerable to adverse outcomes from infection were designated “clinically extremely vulnerable” and advised to “shield.” This involved prolonged confinement at home with strict limits on face-to-face contact, beyond national restrictions. Shielding ended in September 2021 and was considered likely to have harmed mental health and wellbeing. As the UK moved toward a new phase of “living with COVID-19” the mental health and wellbeing experiences of those advised to shield may have diverged from the general population.

**Methods:**

This study is a secondary analysis of nine “COVID-19 Survey” waves of Understanding Society, a longitudinal study of UK participants covering April 2020 to September 2021 alongside pre-pandemic baseline data. The prevalence of clinically significant psychological distress (General Health Questionnaire 12) and low life satisfaction were examined at each wave for participants with longitudinal responses across all waves, stratified by receipt of shielding guidance (Received *n* = 410, Not received *n* = 6,878). Mixed effects regression modeling examined associations between shielding guidance receipt and mental health and life satisfaction when adjusting for potential confounders including age and sex, pre-pandemic mental health/life satisfaction, and loneliness.

**Results:**

Those who received shielding guidance were more likely to experience poor mental health and low life satisfaction during the pandemic. However, this largely reflected differences in pre-pandemic baselines. Variation between waves broadly coincided with the changing burden of COVID-19 and associated restrictions, with similar patterns regardless of shielding guidance receipt. Regression modeling combining data across all waves indicated that receipt of shielding guidance did not independently predict adverse outcomes. However, poor pre-pandemic mental health and low life satisfaction, and frequent loneliness, as well as demographic factors including sex and age, consistently predicted adverse pandemic mental health and wellbeing.

**Discussion:**

While those who received shielding guidance did on average experience poorer mental health and life satisfaction during the pandemic, this study suggests this largely reflects existing inequalities. Drawing on data throughout the shielding program, it addresses an existing evidence gap. These findings reinforce the importance of addressing existing mental health inequalities in the recovery from the current pandemic and for future preparedness.

## 1. Introduction

With the onset of the COVID-19 pandemic in March 2020, the UK Government implemented measures in England to reduce the spread of the virus and to protect those considered most vulnerable. Those with pre-existing medical conditions considered to substantially increase the risk of poor outcomes from COVID-19 were designated as “clinically extremely vulnerable” (CEV) and advised to remain at home and strictly avoid contact with anyone outside of their household, leaving only for essential healthcare, an approach termed “shielding” (1). Initially including around 2.2 million people in England (3.9% of the population), the CEV criteria evolved during the pandemic and expanded significantly in February 2021 to include around 3.7 million (6.6% of the population) (2, 3). The devolved nations of Scotland, Wales and Northern Ireland implemented very similar measures (4–6).

The “Shielded patient list” was created by NHS Digital to identify those who were clinically extremely vulnerable based on the current criteria set out by the United Kingdom Chief Medical Officers (1). This combined identification of prespecified high risk conditions coded to a patient’s medical record with those patients individually identified by their General Practice or hospital specialist as being at high risk. This was maintained as a live list, which was updated weekly between March 2020 to September 2021 according to evolving CEV criteria and to reflect the current circumstances of individual patients. Shielding guidance was sent by NHS England through letters and text messages. Recognizing that this process was incomplete, general practices were requested by NHS England to review their patient lists and identify and contact any patients that should be included and equally to respond to any patients who had incorrectly been identified as having to shield (7).

Shielding restrictions ran alongside national lockdowns but with more restrictive measures and continued beyond the easing of measures in the general population (7, 8). The shielding guidance also evolved during the pandemic, generally becoming less restrictive. It was temporarily paused (re-aligning with guidance for the general population) in August 2020 after the first wave as case numbers fell (9). Shielding guidance was strengthened again (to stay at home with the exception of exercise and medical appointments) with the introduction of the second lockdown in November 2020 and reinstated again with the third lockdown in January 2021 before being gradually relaxed and was withdrawn completely in September 2021. By this point, the vaccination program had achieved extensive coverage in the population and those previously designated CEV were informed that their risk was now broadly in line with the general population (10). Prior to its cessation, there was concern about the negative impacts of shielding on mental health and wellbeing (11).

Large observational studies conducted during the pandemic have produced relatively consistent findings regarding measures of mental health in the UK. Higher levels of anxiety and depressive symptoms were observed in the population during the initial lockdown though these reduced as cases fell, even before the first lockdown eased, prior to rising again during the second wave (12–16). Frequent sampling of the UK Office for National Statistics (ONS) personal wellbeing measures revealed similar trends (17).

Several mixed methods studies (18–20), cross-sectional studies (21–24), and qualitative studies (25–28) have examined mental health and wellbeing experiences associated with shielding in groups with chronic health conditions (18, 21, 28) or specific CEV groups (19, 22–25). Most found that shielding during the pandemic had been harmful to mental health and wellbeing for many participants (18, 20, 21, 24) or more generally for quality of life (22). Shielding may have contributed to higher levels of social isolation (24, 28, 29), which is considered to have a negative impact on mental health and wellbeing (30). Disrupted and changed forms of social contact may also have impacted feelings of isolation and loneliness (29). It has been suggested that access to outdoor green space may have moderated negative feelings associated with confinement (31). Conversely, reducing the risk of contracting COVID-19 by following shielding advice (32) may have been protective for mental health and wellbeing, although receiving the guidance may have heightened their perceived vulnerability to the virus (21). Additionally, some positive impacts on wellbeing have been suggested, possibly linked to slower paced lifestyles during lockdowns (19).

The ONS Shielding Behavioral Survey recruited around 4,000 people designated as CEV to survey at frequent intervals in order to better understand shielding behavior and impacts, and so to inform policy making (33). Around a third reported worsening mental health during the initial months of the pandemic with similar deteriorations reported with the second wave in January 2021.

Longitudinal studies indicate that those who would have been anticipated to be shielding during the pandemic, may have experienced poorer mental health than the general population (32, 34–36). Analysis of data from the English Longitudinal Study of Ageing found that those reporting shielding had significantly increased odds of elevated depressive symptoms when adjusting for a range of potential confounders (35). An analysis of data from Understanding Society data from the first four waves found an association of elevated General Health Questionnaire 12 (GHQ-12) scores with CEV status but not with shielding letter receipt (32). However, this relationship may have changed during the course of the pandemic (27, 28, 32) and it has also been suggested that the relaxation of shielding restrictions may in itself have been experienced negatively, through the loss of these protections against the virus (37, 38), warranting further examination.

This study was designed to build on current understanding of the risks (or potential benefits) to mental health and wellbeing from issuing shielding guidance. This study aimed to extend analysis of longitudinal data on mental health and wellbeing further into the pandemic. Specifically, it examined whether measures of mental health and wellbeing changed for those advised to shield, the extent to which any differences varied from those not advised to shield, and the extent to which the receipt of shielding guidance might have been an independent risk or protective factor for poor mental health and life satisfaction in the longer term.

Although shielding guidance has formally ended, it remains important to identify learning to inform preparedness planning for future pandemics. Even during the COVID-19 pandemic and post-pandemic period, shielding could be considered again if new variants with high lethality and vaccine escape emerge (10). Additionally, with growing evidence that public mental health and wellbeing deteriorated during this period, identifying those most likely to have experienced worsening of their mental health and wellbeing will inform how resources can be targeted most equitably and efficiently (39).

## 2. Methods

### 2.1. Data source

This study is a secondary analysis of longitudinal (panel) data from Understanding Society (the UK Household Longitudinal Survey) COVID-19 survey (40). This established longitudinal study has produced annual data from a representative sample of the UK population and covers health, attitudes, work, and social life (40). The additional COVID-19 online survey ran monthly from April to July 2020 then bi-monthly to March 2021 with a final survey in September 2021 (41). Some questionnaire items were repeated every wave while others were included intermittently (42).

**Figure 1:**
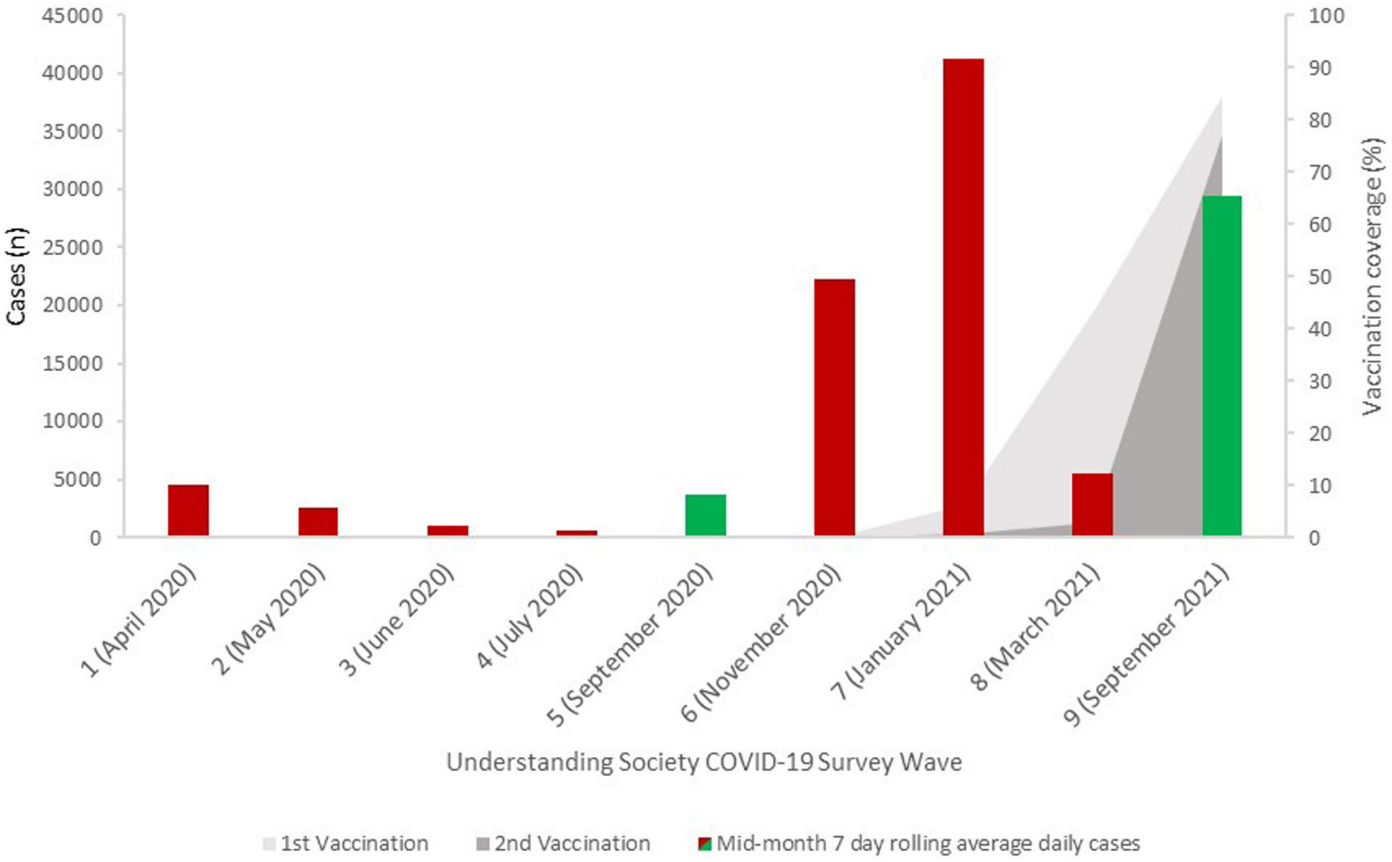
COVID-19 cases and vaccination rates in England during the study period [Source: UK Government]. Red bars show periods of shielding restrictions and green when shielding restrictions were paused. In November 2020 a national lockdown was in place with additional guidance for people considered CEV but this fell short of full shielding restrictions.

The timing of the COVID-19 survey waves covers the full period of the shielding program in England. At various points during the pandemic, there were nationwide lockdowns (stay at home orders for all) which meant that whole population was similarly restricted. There were also periods when lockdown restrictions had eased but shielding guidance remained in place. This is important in recognizing that at some points, the level of restriction was high for the whole population, while at other points there was a greater disparity in the restriction experienced by those following shielding guidance compared to the rest of the population. In addition, the wider context of the pandemic in terms of the relative peaks and troughs in new cases in England (43), as well as the introduction of the vaccination programs in relation to the timing of the survey waves is summarized in Figure 1.

### 2.2. Participants

All participants in Waves 1–9 of the COVID-19 survey of Understanding Society were eligible for inclusion. No data was drawn from the youth survey which ran along this in some waves. All survey participants were ≥16 years of age.

Exclusion criteria were applied for missing exposure or outcome measures in any study wave. This produced a balanced panel with complete longitudinal exposure and outcome data across all study waves including a pre-pandemic baseline, to allow more reliable examination of longitudinal changes.

### 2.3. Measures

#### 2.3.1. Exposure

Self reported receipt of a shielding letter was recorded in Waves 1–5 and repeated in Waves 7–8 following updated definitions (44). The main analysis uses the original shielding definitions. The updated definitions produced a larger exposed participant group, and this was used in a sensitivity analysis. Participants who reported not having received a shielding guidance letter formed the comparator group.

The exposure should be considered as “perceived receipt,” reflecting recall of receiving the letter and recognizing it as guidance to shield. It has previously been found that receipt of a shielding letter strongly predicted shielding behavior (45) though letters were not always received (46).

#### 2.3.2. Outcomes

##### 2.3.2.1 Primary outcome

The primary outcome was mental health as measured by the GHQ-12, a self-administered tool to assess psychological distress and detect common (also termed non-psychotic) mental disorder (47). It has been validated against the Composite International Diagnostic Interview, a gold standard structured interview to identify mental health problems according to commonly used diagnostic classifications (48).

There are 12 questions, each a 4-point Likert scale producing a total score of 0–36. In addition, it is presented as “caseness,” where each question is scored as a binary (for ≥2 in each question) to give a score 0–12 (47). Caseness ≥4 is indicative of “clinically significant psychological distress” (36, 47) and reflects likely presence of a common mental disorder (32, 47). Binary GHQ-12 caseness was selected as the primary outcome, reflecting the rationale of this study to identify potential differences in mental health need across groups.

##### 2.3.2.2 Secondary outcome

Life satisfaction was selected, reflecting its status as a key measure of mental wellbeing in established studies (49, 50). The included survey question was “How satisfied are you currently with your life overall?.” The response was selected from a 7-point Likert type scale ranging from “Completely dissatisfied” to “Completely satisfied.” From this scale, a binary outcome measure was generated to reflect participants reporting low life satisfaction. In the main analysis, the cut-off for this was from “(1) Completely dissatisfied” to “(3) Somewhat dissatisfied.” In the sensitivity analysis this threshold was increased to include “(4) Neither satisfied nor dissatisfied.”

The other ONS defined Personal Wellbeing measures (happiness, anxiety, worthwhile activities in life) (49) were not measured in the COVID-19 survey nor were there any other measures of self-reported wellbeing. As such, the secondary outcome reflects an assessment of life satisfaction rather than the wider concept of wellbeing.

#### 2.3.3 Covariates

Potential confounding factors including demographic characteristics (age, sex, and ethnicity) were adjusted for in regression analysis. COVID-19 vulnerability including CEV status was available as a derived variable. This was based on a series of items where participants self-reported medical conditions which made up the CEV definition criteria, such as severe asthma or chronic obstructive pulmonary disease, some cancers, and some hematological, or solid organ transplants (51). A further mutually exclusive category of “moderate risk/clinically vulnerable” was also included and this included a wider range of risk factors such as being aged 70 years or older, being pregnant, having a specified chronic condition (including diabetes or chronic kidney disease), or having a condition that means they are at higher risk of getting infections. Separately, an adjustment was also made for a different self-rated measure of perceived risk of catching COVID-19. In addition, factors linked to the experience of isolation including whether the person had a partner, lived alone, had access to outdoor space,^1^ and how often they experienced loneliness were adjusted for. Finally, baseline mental health (or wellbeing), recorded from the most recent pre-pandemic survey wave using the same measure, was included in the relevant analyses to account for existing differences between groups at baseline.

### 2.6. Data analysis

Stata 17-SE (52) was used for all analyses. Participants who had not responded to one or more of the survey waves (unit missing) or with incomplete exposure or outcome data in any wave (item missing) were excluded from analysis to create a balanced panel of participants with complete longitudinal response.

Descriptive statistics (number and percentage, and median and inter quartile range) were calculated to summarize the baseline characteristics of participants according to exposure status at entry to Wave 1.

#### 2.6.1. Mental health and wellbeing measure by wave

Descriptive statistics were used to examine mental health and wellbeing (outcome) measures for the exposed and unexposed group at each study wave (including the baseline). Point estimates were plotted (with their 95% confidence intervals) to examine both differences by wave and also between exposed and unexposed groups. Unweighted and weighted samples were analyzed.

#### 2.6.2. Regression modeling

To further explore any association between exposure and outcome variables, regression modeling was used to adjust for covariates hypothesized to be potential confounders or moderators. Logistic regression was used for data from individual waves. Mixed effects logistic regression was used when combining data from all waves. This generates fixed effects estimates for each independent variable and accounts for clustering at the participant level across survey waves. Outputs for the fixed effects component were expressed as odds ratios with 95% confidence intervals, and *p*-values interpreted at the 5% significance level.

A series of sensitivity analyses were developed to test the robustness of findings when differing approaches to analysis were used, including changing binary thresholds of outcomes and analysing GHQ-12 as a continuous outcome using mixed effects linear regression. Additional weighted analysis, using longitudinal weights produced by the data custodians, was undertaken to account for the complex sampling and to generate findings generalizable to the UK population.

### 2.7. Ethics statement

Data from all COVID-19 survey waves were available via the UK Data Service and included a baseline dataset from the two most recent waves of the UKHLS (53). Data are “Safeguarded” and were accessed under the standard End User License agreement and used in accordance with this (54). Understanding Society and the COVID-19 survey have received ethical approval from University of Essex Ethics Committee. For this secondary analysis, no additional ethical approval was required.

## 3. Results

### 3.1. Participant baseline characteristics

There were 7,288 participants with complete longitudinal data for exposure and outcome measures, reflecting a longitudinal participant response rate of 38%. Excluding participants with any missing data for the control variables (complete case analysis in adjusted regression modeling) resulted in the inclusion of 7,085 participants (97% of the total sample of longitudinal responders).

Baseline characteristics of the participants are summarized in Table 1. Those receiving the shielding letter were on average older, with median age 66 (IQR 56–72) vs. 58 (IQR 45–67). There was a higher proportion of males (50.2% vs. 41.4%) while ethnicity was broadly similar. Those receiving a shielding letter slightly more frequently lived alone or were single, also reporting loneliness slightly more frequently.

**Table 1:** Baseline characteristic for participants stratified by exposure status.

	Shielding letter received?			
	No (*n* = 6,878)		Yes (*n* = 410)	
	Median	IQR	Median	IQR
**Age (years)**	58	45.67	66	56.72
**Age group (years)**				
<25	247	3.6%	7	1.7%
25–34	489	7.1%	12	2.9%
35–44	878	12.8%	17	4.1%
45–54	1,318	19.2%	58	14.1%
55–64	1,720	25.0%	96	23.4%
65–74	1,655	24.1%	151	36.8%
>74	571	8.3%	69	16.8%
Missing	0	–	0	–
**Sex**	***n***	**%**	***n***	**%**
Male	2,850	41.4%	206	50.2%
Female	4,028	58.6%	204	49.8%
Missing	0	–	0	–
**Ethnicity**				
Any white background	6,369	93.0%	384	94.1%
Any non-white background	482	7.0%	24	5.9%
Missing	27	–	2	–
**COVID-19 vulnerability**				
No vulnerability	4,129	60.3%	38	9.3%
Moderate risk – clinically vulnerable	2,444	35.7%	181	44.3%
High risk – clinically extremely vulnerable	275	4.0%	190	46.5%
Missing	30	–	1	–
**Perceived risk of catching COVID-19** ^a^				
Very likely	33	0.5%	6	1.5%
Likely	312	4.6%	9	2.2%
Unlikely	4,077	59.5%	207	50.6%
Very unlikely	2,429	35.5%	187	45.7%
Missing	27	–	1	–
**Living with partner**				
Not living with partner	1,757	25.5%	123	30.0%
Living with partner	5,121	74.5%	287	70.0%
Missing	0	–	0	–
**Living alone** ^a^				
No	5,701	82.9%	317	77.3%
Yes	1,177	17.1%	93	22.7%
Missing	7	–	0	–
**Loneliness**	***n***	**%**	***n***	**%**
Hardly ever or never	4,686	68.1%	246	60.0%
Some of the time	1,790	26.0%	123	30.0%
Often	401	5.8%	41	10.0%
Missing	2	–	0	–
**Access to some outdoor space**				
No – Not mentioned/missing^b^	157	2.3%	11	2.7%
Yes – Mentioned	6,721	97.7%	399	97.3%
Missing	–	–	–	–

IQR, Interquartile range.

a “COVID-19 risk perception” and “Living alone” were first sampled in Wave 2. These values are carried back to Wave 1 for the purpose of the baseline table only.

b “No” includes not mentioned and missing. “Yes” is mentioned only.

As would be expected, the proportion identified as CEV was markedly higher in the group receiving a shielding letter (46.5% vs. 4.0%). However, there were still more participants identified as CEV who did not report receiving a letter (*n* = 275 vs. *n* = 190) than who did. In terms of the perception of the risk of catching COVID-19, a higher proportion of those receiving a shielding letter reported they considered themselves very unlikely to catch it (45.7% vs. 35.5%).

### 3.2. Descriptive statistics

**Figure 2:**
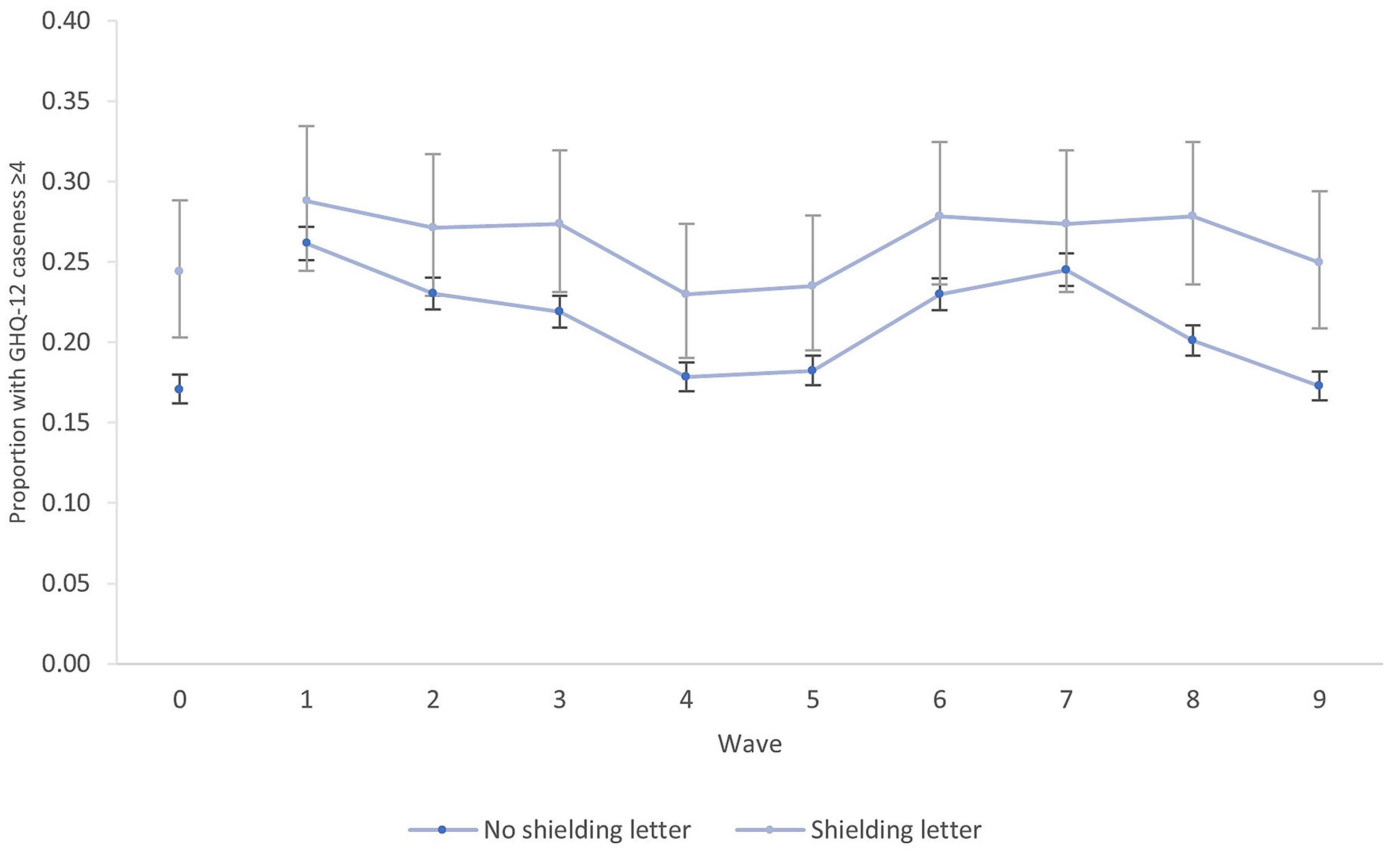
Proportion of participants with GHQ-12 caseness ≥4 (clinically significant psychological distress) with 95% confidence intervals by shielding letter receipt. Wave 0 is pre-pandemic baseline.

**Figure 3:**
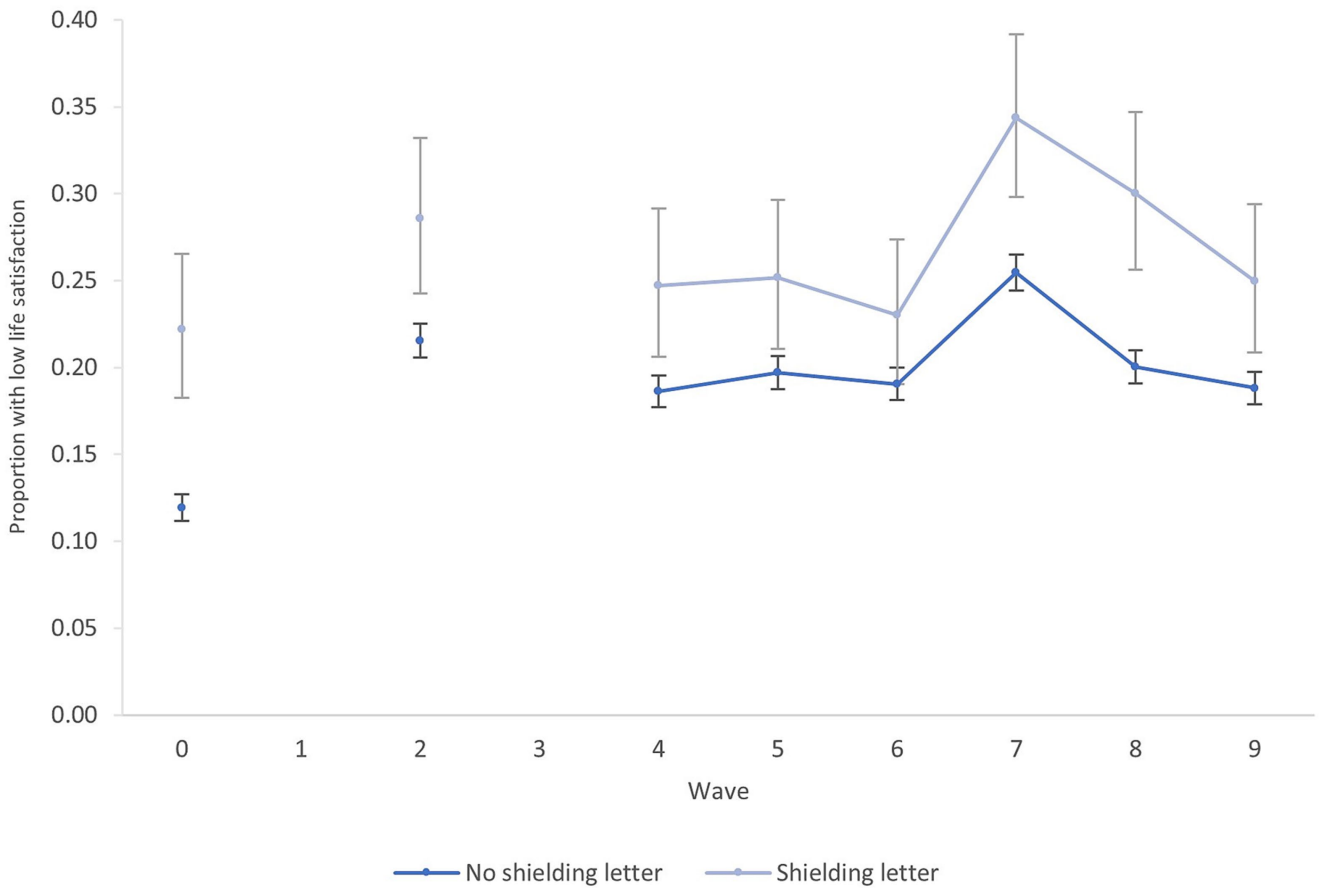
Proportion of participants low life satisfaction with 95% confidence intervals by shielding letter receipt. Wave 0 is pre-pandemic baseline.

Figure 2 shows primary outcomes and Figure 3 shows secondary outcomes over each study wave, stratified by exposure status. For each outcome there is variation over time with similar higher proportions with adverse outcomes at the start of the study period, which then declines before rising again during the survey waves. The pattern broadly coincide with the period of resurgent cases and renewed shielding guidance. Outcomes are improving again by the end of the study period, which coincides with the end of shielding guidance and the rapid progress toward vaccination.

Clinically significant psychological distress and low life satisfaction occurred more frequently in the group reporting receipt of a shielding letter. These differences were statistically significant at the 5% level for some waves, where confidence intervals for point estimates did not overlap. However, statistically significant differences also existed in the baseline measures between groups advised to shield vs. not.

The exposed and unexposed cohorts largely track each other. There is some divergence in the final two waves in the primary outcome indicating that there remained a relative slightly higher proportion of those in the exposed group with clinically significant psychological distress, though proportions in both groups are declining.

### 3.3. Regression modeling

In the unadjusted analysis (Table 2), receipt of a shielding letter was associated with moderately increased odds of clinically significant psychological distress in each wave. Point estimates were similar across waves with substantial overlap within 95% confidence intervals of each wave. Combining data from all waves, participants who received a shielding letter had higher odds of experiencing clinically significant psychological distress (OR 1.50, 95% CI 1.14, 1.97, *p* = 0.004). However, in the fully adjusted analysis, no significant association was found between the exposure variable and clinically significant psychological distress in any individual wave or the combined dataset, refuting an independent association.

**Table 2:** Unadjusted and adjusted odds ratios from logistic regression models for each outcome for those who received a shielding letter compared to those who did not receive a shielding letter by study wave.

Wave	GHQ-12 caseness ≥4		Low life satisfaction	
	Unadjusted OR	Adjusted OR	Unadjusted OR	Adjusted OR
1	1.14 (0.92–1.42)	–	–	–
2	1.24 (0.99–1.56)	1.06 (0.79–1.41)	1.46 (1.17–1.82)	1.03 (0.79–1.33)
3	1.35 (1.08–1.68)	1.00 (0.73–1.36)	–	–
4	1.38 (1.09–1.75)	1.09 (0.79–1.52)	1.43 (1.14–1.81)	1.10 (0.83–1.47)
5	1.38 (1.09–1.74)	0.93 (0.67–1.30)	1.37 (1.09–1.73)	0.86 (0.64–1.15)
6	1.29 (1.04–1.62)	0.88 (0.65–1.20)	1.27 (1.00–1.61)	0.90 (0.67–1.20)
7	1.16 (0.93–1.45)	0.88 (0.65–1.19)	1.53 (1.24–1.89)	1.10 (0.84–1.43)
8	1.53 (1.23–1.92)	1.19 (0.87–1.61)	1.71 (1.38–2.13)	1.20 (0.92–1.58)
9	1.59 (1.26–2.01)	1.14 (0.82–1.57)	1.43 (1.14–1.81)	0.95 (0.72–1.26)

This was similar for life satisfaction with participants in the exposed cohort having higher odds of low life satisfaction in the unadjusted analysis (OR 1.79, 95% CI 1.43, 2.24, *p* < 0.001). In the fully adjusted analysis, shielding letter receipt no longer showed a statistically significant association distress in any individual wave (Table 2) or the combined dataset with life satisfaction.

Examining the other control variables in the full regression model for the primary outcome (Table 3) and secondary outcome (Table 4) revealed significant associations between all outcomes and demographics (age group and sex), their baselines values, loneliness, and some survey waves.

**Table 3:** Output from mixed effects linear regression models combining available data from all study waves with odds ratios for GHQ caseness ≥4 (clinically significant psychological distress).

Variable		OR	95% CI	*p*-value
Shielding letter received?	No	1.00	–	–
	Yes	1.08	(0.84–1.41)	0.543
Age group (years)	<25	0.63	(0.45–0.89)	0.008
	25–34	0.71	(0.56–0.90)	0.004
	35–44	1.03	(0.85–1.23)	0.008
	45–54	1.00	–	–
	55–64	0.79	(0.67–0.92)	0.003
	65–74	0.59	(0.50–0.71)	<0.001
	>74	0.61	(0.48–0.78)	<0.001
Sex	Male	1.00	–	–
	Female	1.98	(1.76–2.24)	<0.001
Ethnicity	White	1.00	–	–
	Non-white	0.66	(0.53–0.83)	<0.001
High GHQ-12 caseness at baseline	No	1.00	–	–
	Yes	7.67	(6.65–8.85)	<0.001
COVID-19 risk perception	Very likely	1.00	–	–
	Likely	1.00	(0.66–1.51)	0.989
	Unlikely	0.81	(0.54–1.21)	0.303
	Very unlikely	0.66	(0.44–0.98)	0.042
COVID-19 vulnerability	Low	1.00	–	–
	Moderate	0.96	(0.85–1.08)	0.493
	CEV	1.25	(1.02–1.54)	0.031
Living alone	No	1.00	–	–
	Yes	0.95	(0.79–1.14)	0.593
Partner	Yes	1.00	–	–
	No	1.00	(0.85–1.19)	0.970
Loneliness (pandemic)	Never	1.00	–	–
	Some of the time	7.62	(7.01–8.29)	<0.001
	Often	59.84	(50.34–71.12)	<0.001
Private outdoor space	Yes	1.00	–	–
	No	1.00	(0.70–1.43)	0.996
Wave	1	–	–	–
	2	1.00	–	–
	3	0.92	(0.83–1.03)	0.152
	4	0.56	(0.50–0.63)	<0.001
	5	0.56	(0.50–0.63)	<0.001
	6	0.85	(0.76–0.95)	0.005
	7	0.90	(0.81–1.01)	0.067
	8	0.67	(0.60–0.75)	<0.001
	9	0.55	(0.49–0.62)	<0.001

**Table 4:** Output from mixed effects linear regression models combining available data from all study waves with odds ratios for low life satisfaction.

Variable		OR	95% CI	*p*-value
Shielding letter received?	No	1.00	–	–
	Yes	1.11	(0.90–1.38)	0.329
Age group (years)	<25	0.38	(0.27–0.52)	<0.001
	25–34	0.43	(0.34–0.53)	<0.001
	35–44	0.70	(0.59–0.83)	<0.001
	45–54	1.00	–	–
	55–64	1.16	(1.01–1.33)	0.037
	65–74	1.01	(0.87–1.17)	0.913
	>74	1.00	(0.82–1.22)	0.971
Sex	Male	1.00	–	–
	Female	0.90	(0.81–0.99)	0.034
Ethnicity	White	1.00	–	–
	Non-white	1.19	(0.98–1.44)	0.075
Low life satisfaction at baseline	No	1.00	–	–
	Yes	5.32	(4.62–6.13)	<0.001
COVID-19 risk perception	Very likely	1.00	–	–
	Likely	1.10	(0.75–1.62)	0.614
	Unlikely	0.88	(0.61–1.27)	0.503
	Very unlikely	0.86	(0.59–1.24)	0.413
COVID-19 vulnerability	Low	1.00	–	–
	Moderate	1.08	(0.97–1.21)	0.146
	CEV	1.04	(0.86–1.25)	0.706
Living alone	No	1.00	–	–
	Yes	0.84	(0.71–1.00)	0.047
Partner	Yes	1.00	–	–
	No	1.39	(1.19–1.62)	<0.001
Loneliness (pandemic)	Never	1.00	–	–
	Some of the time	2.37	(2.19–2.57)	<0.001
	Often	9.27	(7.97–10.77)	<0.001
Private outdoor space	Yes	1.00	–	–
	No	0.70	(0.51–0.97)	0.031
Wave	1	–	–	–
	2	1.00	–	–
	3	–	–	–
	4	0.78	(0.71–0.86)	<0.001
	5	0.85	(0.77–0.94)	0.001
	6	0.74	(0.67–0.82)	<0.001
	7	1.28	(1.16–1.41)	<0.001
	8	0.86	(0.78–0.95)	0.003
	9	0.79	(0.72–0.88)	<0.001

Loneliness showed the strongest association with all adverse outcomes. For clinically significant psychological distress, odds were estimated to be 59.8 times higher (95% CI 50.3–71.1, *p* < 0.001) for those experiencing loneliness often than those reporting never experiencing it.

Poor mental health and life satisfaction at baseline strongly predicted adverse outcomes during the pandemic waves, with odds 7.67 times higher for clinically significant psychological distress (95% CI 6.65–8.85 times higher, *p* < 0.001).

CEV status was also associated with moderately increased odds of adverse outcomes for clinically significant psychological distress (OR 1.25, 95% CI 1.02–1.54) compared to the low risk group. However, there was little difference between those in the low and moderate vulnerability groups. For life satisfaction, no significant association was found.

Lower perception of the risk of catching COVID-19 was associated with statistically significant lower odds of clinically significant psychological distress when comparing the highest and lowest levels.

Female sex was associated with 1.98 times higher odds of clinically significant psychological distress (95% CI 1.76–2.24) though slightly lower odds of low life satisfaction (OR 0.90, 95% CI 0.81–0.99). Those in middle-aged groups generally had higher odds of adverse outcomes. Only younger age groups (<35 years) had lower odds across all outcomes, with older age groups showing lower odds of clinically significant psychological distress but similar odds of low life satisfaction. Non-white ethnicity was associated with significantly lower odds of clinically significant psychological distress (OR 0.66, 95% CI 0.53–0.83) but no difference for life satisfaction.

### 3.4. Weighted analysis

When using a weighted analysis, the results were consistent with the primary analysis. In the fully adjusted mixed effects logistic regression models, the odds ratios for the exposure variable were closer to the null (Supplementary material). While the use of weighted analysis is acknowledged to be important in generating findings generalizable to the target population, in this case it did not alter the conclusions.

### 3.5. Sensitivity analyses

No substantive differences were found through sensitivity analyses. For GHQ-12 score analyzed by mixed effects linear regression, receipt of a shielding letter was on average associated with a 1.01 point increase in GHQ-12 score (95% CI 0.55, 1.47, *p* = <0.001) in the unadjusted analysis. However, as with the logistic regression models, no significant association was identified in the fully adjusted analysis.

When updated CEV definitions were used, an additional 105 exposed participants from Wave 5 and a further 101 from Wave 7 were included. The unadjusted odds ratios for all outcomes were closer to the null. There were no substantive differences in the adjusted analysis to the main exposure definition.

#### 3.5.1. GHQ-12 caseness threshold

No substantive differences were found in either the unadjusted or fully adjusted analyses with changes to the GHQ-12 caseness threshold reduced to ≥3.

#### 3.5.2. Life satisfaction threshold

Changing this threshold resulted in higher unadjusted odds compared to the main analysis (OR 2.34, 95% CI 1.84, 2.96, *p* = <0.001). In the fully adjusted analysis this remained significant with OR (1.25, 95% CI 1.02, 1.55, *p* = 0.034). This suggests that adjustments to the threshold used may alter the findings in a substantive way. However, these odds are elevated only to a small degree and the lower confidence interval is very close to the null suggesting shielding letter receipt remains a poor predictor of low life satisfaction in this analysis.

## 4. Discussion

### 4.1. Main findings

This study found that receipt of a shielding letter was generally associated with increased likelihood of having poor mental health and life satisfaction throughout the first 18 months of the pandemic. However, when other factors were adjusted for, particularly pre-pandemic mental health and life satisfaction, and loneliness, no significant association with shielding letter receipt was found. There is no compelling evidence of a substantial difference between the trajectory of mental health and life satisfaction for those in receipt of a shielding letter compared to those not.

### 4.2. Strengths and limitations

This study drew on a large longitudinal sample of all-age UK adults, in contrast to some other studies which have used specific populations (35). It benefitted from drawing on all waves of the COVID-19 survey to explore longer term associations and temporal trends in mental health and life satisfaction, where existing longitudinal studies identified included data from relatively early in the pandemic only (32, 35, 45). Relevant mental health and wellbeing outcomes were available including a validated measure of mental health symptoms (GHQ-12). The availability of baseline data contextualized any difference observed, a limitation of newly established studies during the pandemic (45).

A limitation of the Understanding Society dataset is the lack of a variable capturing shielding behavior. Therefore, while this study examines the associations with the provision of guidance to shield, it is not able to reliably identify associations with the behavior of shielding itself. Furthermore, there is a discrepancy between the group who were identified as CEV and the group reporting received shielding guidance. Notably, a substantial number of participants identified as CEV did not report receiving a shielding letter. Potential explanations for this include that letters were not sent to or not received by all CEV individuals, that letters were not recalled as such, or that the way that the CEV status derived variable was generated meant a wider group was recorded than were actually identified as needing to shield. While it is not possible to further identify the main explanations for this finding, it is recognized that the process for identifying and informing patients recommended to shield was incomplete and evolved during the program to involve general practices identifying patients as well as central communications from NHS England (1).

A strong causal design, isolating the effects of being advised to shield in a vulnerable group, would have been to compare those who were advised to shield vs. those who were not within the vulnerable group. However, there are clear ethical reasons this study cannot be conducted. In terms of measurement validity, specific wellbeing measures were limited to life satisfaction, and this was only included from Wave 2 with a further gap in Wave 3.

While there were advantages to combining all available waves of data for analysis, this did raise issues regarding response rates over multiple waves. Cumulative attrition over waves meant only 38% of the Understanding Society COVID-19 survey sample had responses for the exposure and outcome variables of interest in all nine waves. Differences in the characteristics of this subset and the main cohort may have led to systematic differences in findings, which could impact the conclusions. Additionally, while this study focussed on adjusting for factors relevant to social isolation there was scope for greater control of other potential confounders including socioeconomic status and employment.

### 4.3. Relationship to other research

Several studies examining longitudinal trends in GHQ-12 using the Understanding Society dataset have been published. Pierce et al. found that receipt of shielding letter increased the probability of having a deteriorating or consistently very poor trajectory for GHQ-12 during Waves 1–6 (36). These differences may primarily reflect methodological differences between a binary outcome and four latent classes, which also consider trajectory. However, Kumari et al., using similar methods to this study also did not find a significant association between receipt of a shielding letter and elevated GHQ-12 caseness in adjusted regression models in Waves 1–4 (32).

In an analysis of the ELSA dataset, significant associations between shielding behavior and depressive symptoms were found (35). This may reflect differences in the way shielding was recorded or in the mental health symptom measures used (PHQ-9 and GAD-7). It may also reflect differences in the study population, with the included cohort being older adults only.

Loneliness emerged as a key predictor of poor mental health and life satisfaction outcomes during the pandemic in this study. Adjusting for loneliness in the ELSA analysis resulted in little change in the identified relationship between shielding and symptoms of depression or anxiety (35). Pre-pandemic studies have suggested that loneliness significantly increases the risk of new onset depression (55) and other common mental health problems (56). During the pandemic, data from 1964 adult participants in the COVID-19 Psychological Wellbeing Study in the UK have showed that rates of loneliness were high and associated with moderately increased odds of depression and emotion regulation difficulties (57).

A consistent finding across multiple studies was the importance of adjusting for baseline scores on mental health and life satisfaction outcomes. While this has been accounted for in large, pre-existing longitudinal studies, it has not been possible in many smaller studies or newly recruited cohorts (45). To a significant extent, poorer outcomes during the pandemic appear to reflect baseline differences and indicate the importance of understanding pre-existing health inequalities. Health states are considered to be dynamic and subject to change over time, influenced by various drivers of health. The concept of state dependence is relevant here and can be summarized as the influence of a previous health state on the likelihood of a future health state. Existing research has identified that state dependence is a significant factor (along with individual unobserved heterogeneity) in understanding the persistence of health states (58). This may have relevance in considering the importance of pre-pandemic mental health and life satisfaction as a predictor of pandemic experiences.

These inequalities are also evident when examining the association between core demographics including age and sex. Middle-aged participants had relatively higher odds of adverse outcomes, as did females. This finding for sex is consistent both with pre-pandemic cross-sectional surveys such as the Adult Psychiatric Morbidity Survey (59) as well as pandemic studies including the UCL COVID-19 Social Study (17). However, that study found symptoms of anxiety and depression to be highest in the youngest age group (18–29) whereas this group had lower adjusted odds than middle aged groups in this study.

Previous research has also suggested an association between CEV status and higher levels of health anxiety and fear of contamination (21, 30). However, shielding was intended to reduce the risk of catching COVID-19, even if the vulnerability remained. Findings from this study suggest that risk perception was similar between exposure groups, though a slightly higher proportion of those in the group advised to shield perceived themselves as very likely to catch COVID-19 despite these additional protections, with higher risk perception predicting poorer outcomes.

Physical activity was not examined in this analysis to avoid over-controlling for the ways that receiving shielding guidance might influence mental health and wellbeing. However, it is acknowledged that this may have important relationships with mental health and wellbeing, as well as shielding. Furthermore, this may have been influenced by the circumstances of the pandemic, while also being characterized by state dependence (60). Systematic review level evidence indicates that higher physical activity was associated with higher wellbeing, and lower levels of symptoms of depression, anxiety and stress during the first year of the pandemic (61). Greater levels of confinement for those following shielding guidance may have influenced levels of physical activity, particularly where this was undertaken in outdoor spaces before the pandemic.

### 4.4. Implications

This study highlights the importance of pre-existing mental health and wellbeing inequalities. The gap in mental health and life satisfaction during the pandemic was comparable, if not smaller, for those advised to shield than it was pre-pandemic. Furthermore, a number of the existing risk factors for poor mental health and wellbeing, such as sex and age differences, continued to show significant associations with outcomes during the pandemic. Recovery from the pandemic should continue to refocus on addressing the wider determinants of mental health and the inequalities associated with them. This is consistent with the UK Government strategy for mental health wellbeing recovery (39).

Loneliness emerged as an important predictor of poor mental health and life satisfaction during the pandemic and may have been more important to those shielding. Existing evidence suggests that loneliness may be causally associated with mental health problems. Addressing loneliness is acknowledged as an important focus of public health and is the subject of an ongoing UK Government strategy published in 2018 (62).

### 4.5. Recommendations for future research

The results of this research show that lower mental health and life satisfaction among clinically extremely vulnerable groups advised to shield during COVID-19 largely reflect pre-pandemic differences. Being advised to shield did not appear to exacerbate inequalities in mental health and life satisfaction. However, future research should aim to use more causal approaches, such as natural experiments or regression discontinuity, to isolate the effects of receiving a shielding letter on mental health and wellbeing. Policymakers in the next pandemic will be better informed about the full costs and benefits of advising vulnerable members of the public to shield.

As the pandemic response in the UK has moved into the phase of “living with COVID-19” (63) it is likely that many of those previously shielding have returned to a much greater degree of normality. However, as the final ONS shielding survey suggested, 7 months after shielding guidance ended there remained a small proportion of this cohort (13%) (64) who were continuing to shield. The prolonged impacts of the pandemic on this subgroup may be more significant and may be a specific target group for further research.

## Data availability statement

Data from the Understanding Society COVID-19 Survey is available from the UK Data Service as a “Safeguarded” dataset and is accessed and used under the Standard End User License. This data can be found at: https://beta.ukdataservice.ac.uk/datacatalogue/studies/study?id=8644 Title: Understanding Society: COVID-19 Study, 2020–2021 Data repository: UK Data Service (Study Number: 8644) University of Essex, Institute for Social and Economic Research (2021). Understanding Society: COVID-19 Study, 2020–2021. [data collection]. 11th Edition. UK Data Service. SN: 8644, http://doi.org/10.5255/UKDA-SN-8644-11.

## Ethics statement

The University of Essex Ethics Committee has approved all data collection on Understanding Society main study, COVID-19 surveys and innovation panel waves, including asking consent for all data linkages except to health records. Requesting consent for health record linkage was approved at Wave 1 by the National Research Ethics Service (NRES) Oxfordshire REC A (08/H0604/124), at BHPS Wave 18 by the NRES Royal Free Hospital & Medical School (08/H0720/60) and at Wave 4 by NRES Southampton REC A (11/SC/0274). Approval for asking consent for health record linkage and for the collection of blood and subsequent serology testing in the March 2021 wave of the COVID-19 study was obtained from London – City & East Research Ethics Committee (21/HRA/0644).

This study is a secondary analysis of the COVID-19 study dataset. Ethical approval was not required for the study involving human participants in accordance with the local legislation and institutional requirements. Written informed consent to participate in this study was not required from the participants of the participants’ legal guardians/next of kin in accordance with the national legislation and the institutional requirements.

## Author contributions

SM conceived the study and developed it as a Masters in Public Health Dissertation submission at University of Birmingham including undertaking the analysis. LK supported the design of the study as dissertation supervisor. JM supported the development of the statistical methods as a dissertation supervisor. SM, LK, and JM subsequently refined the study to submit for publication. SM prepared the draft manuscript. LK and JM edited the manuscript. All authors contributed to the article and approved the submitted version.

## Funding

The publication fee for this article was covered by the University of Birmingham.

## Conflict of interest

This study was undertaken as a Masters dissertation level by SM. This Masters programme was funded by their employer but did not influence the undertaking of this study.

The remaining authors declare that the research was conducted in the absence of any commercial or financial relationships that could be construed as a potential conflict of interest.

## Publisher’s note

All claims expressed in this article are solely those of the authors and do not necessarily represent those of their affiliated organizations, or those of the publisher, the editors and the reviewers. Any product that may be evaluated in this article, or claim that may be made by its manufacturer, is not guaranteed or endorsed by the publisher.

## Supplementary material

The Supplementary material for this article can be found online at: https://www.frontiersin.org/articles/10.3389/fpubh.2023.1235903/full#supplementary-material

## Footnotes

^1^Access to outdoor space showed no significant with primary or secondary outcomes in the fully adjusted models. Additional analysis undertaken during the review process also examined for any interaction between shielding and outdoor space as well as the impact of changing this definition from access to any outdoor space to private outdoor space only. There was no evidence of a significant interaction. Access to private outdoor space was reported commonly by participants in both the exposed and unexposed groups. Adjusting for access to private outdoor space instead of access to any outdoor space did not substantively alter the findings. Access to private outdoor space did not predict mental health or life satisfaction in the fully adjusted models and the original analysis is maintained. Further details of this analysis are available on request.

## References

[1] Digital NHS. Shielded patient list. (2022) Available at: https://digital.nhs.uk/coronavirus/shielded-patient-list (Assessed 18th February 2022).

[2] Digital NHS. Coronavirus shielded patient list open data set, England. (2022). Available at: https://digital.nhs.uk/dashboards/shielded-patient-list-open-data-set#top (Accessed 25th August 2022).

[3] Wise J. Covid-19: extra 1.7 million people in England are asked to shield. BMJ. (2021) 372:n467. doi: 10.1136/bmj.n467 33597119

[4] Scottish Government. Coronavirus (COVID-19): advice for people on the highest risk list. (2022). Available at: https://www.gov.scot/publications/covid-highest-risk/ (Accessed 6th March 2022).

[5] Welsh Government. Guidance on protecting people defined on medical grounds as clinically extremely vulnerable from coronavirus (COVID-19) – Previously known as ‘shielding’. (2022). Available at: https://gov.wales/guidance-protecting-people-defined-medical-grounds-clinically-extremely-vulnerable-coronavirus (Accessed 6th March 2022).

[6] Northern Ireland Government. Coronavirus (COVID-19): guidance for ‘clinically extremely vulnerable’ and ‘vulnerable’ people | nidirect. (2022). Available at: https://www.nidirect.gov.uk/articles/coronavirus-covid-19-guidance-clinically-extremely-vulnerable-and-vulnerable-people (Accessed 6th March 2022).

[7] Runswick-Cole K. A (brief) history of shielding. (2020). Available at: https://www.sheffield.ac.uk/ihuman/news/brief-history-shielding (Accessed 2nd August 2022).

[8] Health Foundation. COVID-19 policy tracker. (2020). Available at: https://covid19.health.org.uk/ (Accessed 5th September 2022).

[9] UK Health Security Agency. COVID-19: guidance on protecting people defined on medical grounds as extremely vulnerable. Department of Health and Social Care. (2020) Available at: https://www.gov.uk/government/publications/guidance-on-shielding-and-protecting-extremely-vulnerable-persons-from-covid-19#full-publication-update-history (Accessed 5th September 2022).

[10] England NHS. Information about the end of the shielding programme and managing the closure of the shielded patient list. (2021). Available at: https://www.england.nhs.uk/coronavirus/publication/information-about-the-end-of-the-shielding-programme-and-managing-the-closure-of-the-shielded-patient-list/ (Accessed 18th February 2022).

[11] Department of Health and Social Care. Press release: shielding programme ends for most vulnerable. (2021). Available at: https://www.gov.uk/government/news/shielding-programme-ends-for-most-vulnerable (Accessed 18 February, 2022).

[12] Daly M Robinson E. Psychological distress associated with the second COVID-19 wave: prospective evidence from the UK household longitudinal study. (2022) Available at: https://psyarxiv.com/8mpxr/10.1016/j.jad.2022.05.025 PMC9091072 35568319

[13] Daly M Sutin A Robinson E. Longitudinal changes in mental health and the COVID-19 pandemic: evidence from the UK household longitudinal study. Psychol Med. (2020) 52:2549–58. doi: 10.1017/S0033291720004432 33183370 PMC7737138

[14] Aknin LB de Neve JE Dunn EW Fancourt DE Goldberg E Helliwell JF. Mental health during the first year of the COVID-19 pandemic: a review and recommendations for moving forward. Perspect Psychol Sci. (2022) 17:915–36. doi: 10.1177/17456916211029964 35044275 PMC9274782

[15] Pierce M Hope H Ford T Hatch S Hotopf M John A. Mental health before and during the COVID-19 pandemic: a longitudinal probability sample survey of the UK population. Lancet Psychiatry. (2020) 7:883–92. 32707037 10.1016/S2215-0366(20)30308-4 PMC7373389

[16] Office for Health Improvement and Disparities. COVID-19 mental health and wellbeing surveillance: report. (2021). Available at: https://www.gov.uk/government/publications/covid-19-mental-health-and-wellbeing-surveillance-report (Accessed 22nd February 2022).

[17] Office for Health Improvement and Disparities. Wider impacts of COVID-19. (2022). Available at: https://analytics.phe.gov.uk/apps/covid-19-indirect-effects/ (Accessed 27th February 2022).

[18] Lasseter G Compston P Robin C Lambert H Hickman M Denford S. Exploring the impact of shielding advice on the health and wellbeing of individuals identified as extremely vulnerable and advised to shield in Southwest England amid the COVID-19 pandemic: a mixed-methods evaluation. medRxiv. (2022). doi: 10.1101/2022.01.05.21268251 PMC9685010 36418978

[19] Sloan M Gordon C Lever E Harwood R Bosley MA Pilling M. COVID-19 and shielding: experiences of UK patients with lupus and related diseases. Rheumatol Adv Pract. (2021) 5:rkab003. doi: 10.1093/rap/rkab003 33728396 PMC7928599

[20] Spurr L Tan HL Wakeman R Chatwin M Hughes Z Simonds A. Psychosocial impact of the COVID-19 pandemic and shielding in adults and children with early-onset neuromuscular and neurological disorders and their families: a mixed-methods study. BMJ Open. (2022) 12:e055430. doi: 10.1136/bmjopen-2021-055430 PMC8968110 35354630

[21] Daniels J Rettie H. The mental health impact of the COVID-19 pandemic second wave on Shielders and their family members. Int J Environ Res Public Health. (2022) 19:7333. doi: 10.3390/ijerph19127333 35742580 PMC9223363

[22] Harris RJ Downey L Smith TR Cummings JRF Felwick R Gwiggner M. Life in lockdown: experiences of patients with IBD during COVID-19. BMJ Open Gastroenterol. (2020) 7:e000541. doi: 10.1136/bmjgast-2020-000541 PMC7677865 33214234

[23] Ramasamy K Sadler R Jeans S Varghese S Turner A Larham J. COVID symptoms, testing, shielding impact on patient-reported outcomes and early vaccine responses in individuals with multiple myeloma. Br J Haematol. (2022) 196:95–8. doi: 10.1111/bjh.17764 34341984 PMC8444854

[24] Westcott KA Wilkins F Chancellor A Anderson A Doe S Echevarria C. The impact of COVID-19 shielding on the wellbeing, mental health and treatment adherence of adults with cystic fibrosis. Future Healthc J. (2021) 8:e47. doi: 10.7861/fhj.2020-0205 33791475 PMC8004337

[25] Antoun J Brown DJ Jones DJW Sangala NC Lewis RJ Shepherd AI. Understanding the impact of initial COVID-19 restrictions on physical activity, wellbeing and quality of life in shielding adults with end-stage renal disease in the United Kingdom Dialysing at home versus in-Centre and their experiences with telemedicine. Int J Environ Res Public Health. (2021) 18:1–17. doi: 10.3390/ijerph18063144 PMC8002886 33803708

[26] Brooke J Clark M. Older people’s early experience of household isolation and social distancing during COVID-19. J Clin Nurs. (2020) 29:4387–402. doi: 10.1111/jocn.15485 32891063

[27] Caton E Chaplin H Carpenter L Sweeney M Tung HY de Souza S. The impact of consecutive COVID-19 lockdowns in England on mental wellbeing in people with inflammatory arthritis. BMC Rheumatol. (2022) 6:37. doi: 10.1186/s41927-022-00266-y 35765098 PMC9241173

[28] Fisher A Roberts A McKinlay AR Fancourt D Burton A. The impact of the COVID-19 pandemic on mental health and well-being of people living with a long-term physical health condition: a qualitative study. BMC Public Health. (2021) 21:1–12. doi: 10.1186/s12889-021-11751-3 34620136 PMC8496145

[29] Hu Y Qian Y. COVID-19, inter-household contact and mental well-being among older adults in the US and the UK. Front Sociol. (2021) 26:143. doi: 10.3389/fsoc.2021.714626 PMC8350320 34381838

[30] Brooks SK Webster RK Smith LE Woodland L Wessely S Greenberg N. The psychological impact of quarantine and how to reduce it: Rapid review of the evidence. Lancet. (2020) 395:912–20. doi: 10.1016/S0140-6736(20)30460-8 32112714 PMC7158942

[31] Aerts R Vanlessen N Honnay O. Exposure to green spaces may strengthen resilience and support mental health in the face of the covid-19 pandemic. BMJ. (2021) 373:n1601. doi: 10.1136/bmj.n1601 34154989

[32] Kumari M Chandola T Booker CL Benzeval MJ. Targeted shielding and coronavirus symptoms among adults in the UK. (2021); Available at: https://www.researchsquare.com

[33] Office for National Statistics. Shielding Behavioural survey (SBS) quality and methodology Information. (2020). Available at: https://www.ons.gov.uk/peoplepopulationandcommunity/healthandsocialcare/healthcaresystem/methodologies/shieldingbehaviouralsurveysbsqmi#important-points (Accessed 22nd February 2022).

[34] Office for National Statistics. Coronavirus and clinically extremely vulnerable people in England - Office for National Statistics. (2021) Available at: https://www.ons.gov.uk/peoplepopulationandcommunity/healthandsocialcare/conditionsanddiseases/bulletins/coronavirusandclinicallyextremelyvulnerablepeopleinengland/18januaryto30january2021#shielding-of-clinically-extremely-vulnerable-people-data (Accessed 6th March 2022).

[35] di Gessa G Price D. The impact of shielding during the COVID-19 pandemic on mental health: evidence from the English longitudinal study of ageing. Br J Psychiatry. (2022):1–7. doi: 10.1192/bjp.2022.44 PMC11920597 35369895

[36] Pierce M McManus S Hope H Hotopf M Ford T Hatch SL. Mental health responses to the COVID-19 pandemic: a latent class trajectory analysis using longitudinal UK data. Lancet Psychiatry. (2021) 8:610–9. doi: 10.1016/S2215-0366(21)00151-6 33965057 PMC9764381

[37] Giles C. Covid-19: for the clinically extremely vulnerable, life hasn’t returned to normal. BMJ. (2022) 376:o397. doi: 10.1136/bmj.o397 35168939

[38] British Red Cross. Lonely and left behind - tackling loneliness at a time of crisis. (2020). Available at: https://www.redcross.org.uk/about-us/what-we-do/we-speak-up-for-change/life-after-lockdown-tackling-loneliness (Accessed 13th March 2022).

[39] Department of Health and Social Care, Cabinet office. COVID-19 mental health and wellbeing recovery action plan - GOV.UK. (2021). Available at: https://www.gov.uk/government/publications/covid-19-mental-health-and-wellbeing-recovery-action-plan (Accessed 2 August, 2022).

[40] Institute for Social and Economic Research at University of Essex. Understanding society – the UK household longitudinal study. (2022). Available at: https://www.understandingsociety.ac.uk/ (Accessed 22nd February 2022).

[41] COVID-19 survey frequently asked questions | Understanding society. (2022) Available at: https://www.understandingsociety.ac.uk/documentation/covid-19/faqs (Accessed 6th March 2022).

[42] Institute for Social and Economic Research (ISER) at the University of Essex. Questionnaires. (2022). Available at: https://www.understandingsociety.ac.uk/documentation/covid-19/questionnaires (Accessed 1st August 2022).

[43] UK Health Security Agency. Coronavirus (COVID-19) in the UK - England summary. (2022) Available at: https://coronavirus.data.gov.uk/ (Accessed 25th August 2022)

[44] Institute for Social and Economic Research at University of Essex. Variable search - understanding society COVID-19 survey. (2022). Available at: https://www.understandingsociety.ac.uk/documentation/covid-19/dataset-documentation (Accessed 24th July 2023).

[45] Bachtiger P Adamson A MacLean WA Kelshiker MA Quint JK Peters NS. Determinants of shielding behavior during the COVID-19 pandemic and associations with well-being among National Health Service Patients: longitudinal observational study. JMIR Public Health Surveill. (2021) 7:e30460. doi: 10.2196/30460 34298499 PMC8454693

[46] Mahase E. Covid-19: Government’s shielding scheme failed thousands of clinically extremely vulnerable people, say MPs. BMJ. (2021) 373:n1033. doi: 10.1136/bmj.n1033 33879475

[47] Jackson C. The general health questionnaire. Occup Med (Chic Ill). (2006) 57:79–9.

[48] Goldberg DP Gater R Sartorius N Ustun TB Piccinelli M Gureje O. The validity of two versions of the GHQ in the WHO study of mental illness in general health care. Psychol Med. (1997) 27:191–7. doi: 10.1017/s0033291796004242 9122299

[49] Office for National Statistics. Personal well-being quarterly estimates technical report. (2019). Available at: https://www.ons.gov.uk/peoplepopulationandcommunity/wellbeing/methodologies/personalwellbeingquarterlyestimatestechnicalreport#seasonal-decomposition-of-quarterly-personal-well-being-data (Accessed 17th February 2022).

[50] Dolan P Laffan K Velias A. Who’s miserable now? Identifying clusters of people with the lowest subjective wellbeing in the UK In: Social Choice and Welfare (2021). 1–32. Available at: https://link.springer.com/article/10.1007/s00355-021-01365-4

[51] Institute for Social and Economic Research at University of Essex. Understanding society COVID-19 user guide. (2021). Available at: https://www.understandingsociety.ac.uk/documentation/covid-19/user-guide (Accessed 13th March 2022).

[52] StataCorp LLC. Stata. (2022). Available at: https://www.stata.com/products/ (Accessed 5th March 2022).

[53] UK Data Service › Series. (2022) Available at: https://beta.ukdataservice.ac.uk/datacatalogue/series/series?id=2000053(Accessed 23rd February 2022).

[54] Types of data access — UK data service. (2022) Available at: https://ukdataservice.ac.uk/help/access-policy/types-of-data-access/ (Accessed 6 March 2022).

[55] Lee SL Pearce E Ajnakina O Johnson S Lewis G Mann F. The association between loneliness and depressive symptoms among adults aged 50 years and older: a 12-year population-based cohort study. Lancet Psychiatry. (2021) 8:48–57. doi: 10.1016/S2215-0366(20)30383-7 33181096 PMC8009277

[56] Mann F Wang J Pearce E Ma R Schlief M Lloyd-Evans B. Loneliness and the onset of new mental health problems in the general population. Soc Psychiatry Psychiatr Epidemiol. (2022) 2022:1–18. doi: 10.1007/s00127-022-02261-7 PMC9636084 35583561

[57] Groarke JM Berry E Graham-Wisener L McKenna-Plumley PE McGlinchey E Armour C. Loneliness in the UK during the COVID-19 pandemic: cross-sectional results from the COVID-19 psychological wellbeing study. PLoS One. (2020) 15:e0239698. doi: 10.1371/journal.pone.0239698 32970764 PMC7513993

[58] Hernández-Quevedo C Jones AM Rice N. Persistence in health limitations: a European comparative analysis. J Health Econ. (2008) 27:1472–88. doi: 10.1016/j.jhealeco.2008.06.007 18687495

[59] NHS Digital. Adult psychiatric morbidity survey - NHS Digital. (2016) Available at: https://digital.nhs.uk/data-and-information/publications/statistical/adult-psychiatric-morbidity-survey (Accessed January 25, 2022).

[60] Kumagai N Ogura S. Persistence of physical activity in middle age: a nonlinear dynamic panel approach. Eur J Health Econ. (2014) 15:717–35. doi: 10.1007/s10198-013-0518-8 23860736 PMC4145203

[61] Marconcin P Werneck AO Peralta M Ihle A Gouveia ÉR Ferrari G. The association between physical activity and mental health during the first year of the COVID-19 pandemic: a systematic review. BMC Public Health. (2022) 22:209. doi: 10.1186/s12889-022-12590-6 35101022 PMC8803575

[62] Department for Digital, Culture, Media & Sport. Government’s work on tackling loneliness (2022). Available at: https://www.gov.uk/guidance/governments-work-on-tackling-loneliness (Accessed 2nd August 2022).

[63] Cabinet Office. COVID-19 response: living with COVID-19. (2022). Available at: https://www.gov.uk/government/publications/covid-19-response-living-with-covid-19 (Accessed 2nd August 2022).

[64] Office for National Statistics. CEV bulletin April 2022. (2022). Available at: https://www.ons.gov.uk/peoplepopulationandcommunity/healthandsocialcare/conditionsanddiseases/bulletins/coronavirusandclinicallyextremelyvulnerablepeopleinengland/4aprilto23april2022 (Accessed 25th July 2022).

