# Supplementary material for: Mental health and life satisfaction among those advised to shield during the COVID-19 pandemic in the UK: a secondary analysis of the Understanding Society longitudinal study
Source: Front Public Health. 2023 Sep 27;11:1235903. doi: 10.3389/fpubh.2023.1235903 (PMC10566375; doi:10.3389/fpubh.2023.1235903)
Supplement: Supplementary file 1 [file Table_1.DOCX]

Supplementary Material

Mental health and wellbeing among those advised to shield during the COVID-19 pandemic in the UK: a secondary analysis of the Understanding Society longitudinal study

Simon George Morris^1,2*^, Laura Kudrna^1^, James Martin^1^

^1^Institute of Applied Health Research, University of Birmingham, Birmingham, United Kingdom

^2^Public Health Specialty Training Programme, NHS England - West Midlands, United Kingdom

*** Correspondence:** Corresponding Author: [georgemorris@nhs.net](mailto:georgemorris@nhs.net)

Table 1: Output from mixed effects linear regression models using longitudinal weights combining available data from all study waves with odds ratios for GHQ caseness ≥4 (clinically significant psychological distress)

| **Variable** |  | **OR** | **95% CI** | **p-value** |
| --- | --- | --- | --- | --- |
| Shielding letter received? | No | 1.00 | - | - |
|  | Yes | 1.00 | (0.76-1.33) | 0.981 |
| Age group (Years) | <25 | 0.59 | (0.41-0.85) | 0.005 |
|  | 25-34 | 0.74 | (0.58-0.94) | 0.014 |
|  | 35-44 | 0.99 | (0.85-1.23) | 0.910 |
|  | 45-54 | 1.00 | - | - |
|  | 55-64 | 0.81 | (0.68-0.96) | 0.013 |
|  | 65-74 | 0.63 | (0.53-0.76) | <0.001 |
|  | >74 | 0.63 | (0.49-0.82) | 0.001 |
| Sex | Male | 1.00 | - | - |
|  | Female | 2.00 | (1.78-2.25) | <0.001 |
| Ethnicity | White | 1.00 | - | - |
|  | Non-white | 0.69 | (0.54-0.88) | 0.003 |
| High GHQ-12 caseness at baseline | No | 1.00 | - | - |
|  | Yes | 6.93 | (5.98-8.04) | <0.001 |
| COVID-19 Risk Perception | Very likely | 1.00 | - | - |
|  | Likely | 1.04 | (0.61-1.76) | 0.889 |
|  | Unlikely | 0.92 | (0.55-1.54) | 0.747 |
|  | Very unlikely | 0.68 | (0.40-1.14) | 0.140 |
| COVID-19 Vulnerability | Low | 1.00 | - | - |
|  | Moderate | 1.02 | (0.89-1.17) | 0.774 |
|  | CEV | 1.40 | (1.06-1.85) | 0.018 |
| Living alone | No | 1.00 | - | - |
|  | Yes | 0.91 | (0.73-1.13) | 0.391 |
| Partner | Yes | 1.00 | - | - |
|  | No | 1.02 | (0.83-1.19) | 0.862 |
| Loneliness (pandemic) | Never | 1.00 | - | - |
|  | Some of the time | 7.37 | (6.51-8.36) | <0.001 |
|  | Often | 53.19 | (40.84-69.28) | <0.001 |
| Private outdoor space | Yes | 1.00 | - | - |
|  | No | 1.02 | (0.71-1.47) | 0.924 |
| Wave | 1 | - | - | - |
|  | 2 | 1.00 | - | - |
|  | 3 | 0.83 | (0.70-0.98) | 0.026 |
|  | 4 | 0.55 | (0.47-0.64) | <0.001 |
|  | 5 | 0.48 | (0.40-0.57) | <0.001 |
|  | 6 | 0.79 | (0.65-0.96) | 0.019 |
|  | 7 | 0.75 | (0.63-0.90) | 0.002 |
|  | 8 | 0.57 | (0.47-0.69) | <0.001 |
|  | 9 | 0.48 | (0.40-0.57) | <0.001 |

Table 2: Output from mixed effects linear regression models using longitudinal weights combining available data from all study waves with odds ratios for low life satisfaction

| **Variable** |  | **OR** | **95% CI** | **p-value** |
| --- | --- | --- | --- | --- |
| Shielding letter received? | No | 1.00 | - | - |
|  | Yes | 1.02 | (0.82-1.26) | 0.880 |
| Age group (Years) | <25 | 0.38 | (0.26-0.56) | <0.001 |
|  | 25-34 | 0.40 | (0.31-0.51) | <0.001 |
|  | 35-44 | 0.68 | (0.56-0.82) | <0.001 |
|  | 45-54 | 1.00 | - | - |
|  | 55-64 | 1.21 | (1.03-1.43) | 0.022 |
|  | 65-74 | 1.06 | (0.90-1.26) | 0.470 |
|  | >74 | 1.08 | (0.85-1.35) | 0.538 |
| Sex | Male | 1.00 | - | - |
|  | Female | 0.90 | (0.81-1.00) | 0.042 |
| Ethnicity | White | 1.00 | - | - |
|  | Non-white | 1.09 | (0.87-1.38) | 0.760 |
| Low life satisfaction at baseline | No | 1.00 | - | - |
|  | Yes | 5.34 | (4.51-6.33) | <0.001 |
| COVID-19 Risk Perception | Very likely | 1.00 | - | - |
|  | Likely | 1.75 | (1.03-2.97) | 0.038 |
|  | Unlikely | 1.29 | (0.77-2.19) | 0.336 |
|  | Very unlikely | 1.24 | (0.73-2.11) | 0.425 |
| COVID-19 Vulnerability | Low | 1.00 | - | - |
|  | Moderate | 1.10 | (0.97-1.21) | 0.146 |
|  | CEV | 1.02 | (0.81-1.27) | 0.894 |
| Living alone | No | 1.00 | - | - |
|  | Yes | 0.82 | (0.62-1.09) | 0.176 |
| Partner | Yes | 1.00 | - | - |
|  | No | 1.40 | (1.11-1.76) | 0.004 |
| Loneliness (pandemic) | Never | 1.00 | - | - |
|  | Some of the time | 2.46 | (2.19-2.75) | <0.001 |
|  | Often | 10.92 | (8.63-13.81) | <0.001 |
| Private outdoor space | Yes | 1.00 | - | - |
|  | No | 0.65 | (0.44-0.98) | 0.040 |
| Wave | 1 | - | - | - |
|  | 2 | 1.00 | - | - |
|  | 3 | - | - | - |
|  | 4 | 0.76 | (0.65-0.88) | <0.001 |
|  | 5 | 0.85 | (0.73-1.00) | 0.043 |
|  | 6 | 0.73 | (0.62-0.85) | <0.001 |
|  | 7 | 1.23 | (1.04-1.45) | 0.014 |
|  | 8 | 0.81 | (0.69-0.95) | 0.011 |
|  | 9 | 0.70 | (0.60-0.81) | <0.001 |
